# Ultrasound Patches Toward Intelligent Theranostics: From Flexible Materials to Closed-Loop Biomedical Systems

**DOI:** 10.3390/bioengineering13030345

**Published:** 2026-03-17

**Authors:** Jinpeng Zhao, Yi Huang, Yuan Zhang, Yuhang Xie, Wei Guo, Yang Li, Shidong Wang

**Affiliations:** 1 School of Clinical Medicine, Peking University Health Science Center, Beijing 100080, China; 2210122420@stu.pku.edu.cn (J.Z.); 2110301230@bjmu.edu.cn (Y.Z.);; 2 Department of Musculoskeletal Tumor, Peking University People’s Hospital, Beijing 100044, China; hyi8363@sina.com (Y.H.); bonetumor@163.com (W.G.); 3 Beijing Key Laboratory of Musculoskeletal Tumor, Peking University People’s Hospital, Beijing 100044, China; 4 Department of General Surgery, The First Medical Center, Chinese PLA General Hospital, Beijing 100039, China

**Keywords:** ultrasound patches, intelligent theranostics, wearable electronic devices, piezoelectric materials, closed-loop therapy

## Abstract

Ultrasound patches represent a transformative advancement beyond conventional ultrasonography, evolving into intelligent theranostic systems for personalized healthcare. This evolution is propelled by synergistic innovations in flexible piezoelectric materials and integrated designs. The development of piezoelectric polymers, lead-free ceramics, and bio-composite materials has laid the foundation for long-term, conformal, and biosafe interfacing with the human body. Structurally, miniaturized transducer arrays (e.g., CMOS-integrated arrays achieving ~200 μm focal spots and 100 kPa focal pressure), multimodal integration, and bioinspired interfaces have enabled high-precision deep-tissue sensing and spatiotemporally controlled energy delivery—exemplified by strain-sensing feedback improving the signal-to-noise ratio by 5 dB for precise neuromodulation. These capabilities are converging to create closed-loop platforms, as demonstrated in continuous cardiovascular monitoring (up to 164 mm depth for 12 h), image-guided neuromodulation for neurological disorders, on-demand drug delivery (achieving 100% higher plasma concentration than ultrasound alone), and integrated tumor therapy with real-time feedback. Despite persistent challenges in material biocompatibility, energy efficiency, and clinical standardization, the future of ultrasound patches lies in their deep integration with multimodal sensing, machine learning, and adaptive control algorithms. This path will ultimately realize their potential for intelligent, closed-loop theranostics in chronic disease management, telemedicine, and personalized therapy.

## 1. Introduction

As the global population ages and the burden of chronic diseases continues to rise [1], there is an unprecedented demand for continuous, dynamic, and personalized health monitoring and diagnostic approaches. In this context, wearable medical electronic devices are rapidly advancing from concept to clinical frontiers [2,3,4], aiming to extend traditional hospital-centered diagnosis and treatment to daily home-based and telemedicine settings. Ultrasound imaging, as a cornerstone of clinical diagnostics, plays a vital role in cardiovascular, oncological, and musculoskeletal fields, among others. However, conventional ultrasound systems are constrained by inherent limitations such as rigid probes, bulky structures, and reliance on liquid coupling agents, which hinder their ability to conform closely to irregular body contours (e.g., joints, thoracic cage) or adapt to tissue deformation during daily activities [5]. Furthermore, they are incapable of supporting long-term continuous monitoring of deep tissue physiology [6,7]. These drawbacks severely restrict their application in emerging scenarios such as telemedicine and chronic disease management.

To overcome these barriers, the “ultrasound patch” has emerged. Its core innovation lies in the use of flexible piezoelectric materials (e.g., polyvinylidene fluoride, silicon nanopillars) and lightweight designs, enabling non-invasive, continuous, and accurate monitoring of deep tissues through conformal skin attachment [5,8,9,10]. For instance, flexible ultrasound transducers (FUSTs) based on silver nanowires and elastic substrates can withstand tensile strains exceeding 110%, weigh only about 1.58 g, and adapt well to highly curved surfaces such as the breast or carotid artery [5]. Ultrasound patch (USoP) systems that incorporate signal acquisition, wireless transmission, and data-processing capabilities have been shown to track physiological signals at depths up to 164 mm continuously for 12 h [7]. Such breakthroughs not only address wearability challenges but also elevate ultrasound from a diagnostic tool to a platform for real-time physiological data streaming.

Building on this foundation, the scope of ultrasound patches is rapidly expanding from diagnosis toward integrated diagnosis and therapy. Their applications now encompass diverse areas, including: home-based autonomous patches for chronic wound treatment [11]; real-time bladder volume monitoring for pediatric neurogenic bladder dysfunction [12]; carotid artery Doppler assessment of cardiovascular function [13,14,15]; sonodynamic therapy for tumors [16]; precise neuromodulation assisting post-stroke motor recovery [17]; and interventions for neurological disorders such as Parkinson’s disease [9,18,19]. This breadth of utility underscores how ultrasound patches are reshaping disease management and treatment paradigms.

This article systematically reviews recent advances in the design and biomedical applications of ultrasound patches. First, we delve into the evolution of piezoelectric and composite materials that define their performance. Next, we examine innovative designs in transducer architecture and array configurations. Finally, we comprehensively present breakthrough applications in disease diagnosis, drug delivery, neuromodulation, and tumor therapy, while also discussing challenges and future directions toward intelligent, integrated diagnostic-therapeutic systems.

## 2. Material Selection

In the design of ultrasound patches, material selection is a core determinant of performance and applicable scenarios, with development trends shifting from pursuing single properties toward a balanced integration of energy conversion efficiency, physical flexibility, and biosafety (Table 1). Early efforts primarily focused on piezoelectric ceramics with high electromechanical conversion efficiency [20] and piezoelectric polymers offering superior flexibility [21]. To address challenges related to biocompatibility and environmental sustainability, lead-free piezoelectric materials and natural biomaterials (e.g., chitosan) [22] have been extensively explored. Concurrently, composite material systems that integrate the advantages of the above mentioned materials have further expanded the application boundaries of ultrasound patches [23].

Piezoelectric polymers are among the commonly used materials in ultrasound patch design. Polyvinylidene fluoride (PVDF) and its co-polymers have been widely studied owing to their excellent piezoelectric properties and flexibility. AlMohimeed and Ono [24] developed a wearable ultrasound sensor (WUS) based on a bilayer PVDF piezoelectric polymer film. Fabricated via a simple, low-cost process, the sensor is flexible, lightweight, thin, and compact, enabling secure attachment to the skin without affecting the contraction dynamics of the target muscle. They employed this WUS to monitor contractions of the human gastrocnemius muscle. Parameters such as maximum contraction thickness and contraction time were extracted, demonstrating the value of PVDF-based ultrasound sensors for low-cost, noninvasive, and continuous monitoring of skeletal muscle contraction characteristics. Furthermore, Liu and Wu [25] developed a flexible piezoelectric micromachined ultrasonic transducer (PMUT) using silver-coated PVDF film mounted on a laser-processed polymeric substrate via low temperature (<100 °C) bonding. This PMUT can conform to flat, concave, and convex surfaces while maintaining good acoustic performance, further highlighting the significant potential of PVDF in ultrasound and wearable device applications.

Piezoelectric ceramic materials, known for their high piezoelectric coefficients and favorable electromechanical coupling properties, are employed in ultrasound patches requiring high sensitivity and resolution. Joshi et al. [26] reported a flexible, row-column addressed PMUT array that uses lead zirconate titanate (PZT) thin film as the active piezoelectric layer and polyimide as the passive layer. After optimization, the array is suitable for wearable devices (e.g., health monitoring) or ultrasonic detection in shallow water environments. Song et al. [27] integrated high-performance Pb(Mg_1/3_Nb_2/3_)O_3_-Pb(Zr,Ti)O_3_ (PMN-PZT) piezoelectric ceramic with a flexible polydimethylsiloxane (PDMS) substrate. When attached to the skin, the resulting device enables real-time monitoring of bone condition.

However, lead-containing materials pose certain biotoxicity risks. Although the aforementioned studies employed polymer encapsulation to prevent direct skin contact with lead and emphasized use only in superficial wearables, the adoption of lead-free materials remains a preferable choice. Wang et al. [28] fabricated a hybrid nanopatch using the lead-free piezoelectric material barium titanate-reduced graphene oxide (BTO/rGO), which successfully promoted the differentiation of neural stem cells into functional neurons and demonstrated significant efficacy in the treatment of traumatic brain injury. By avoiding the toxicity associated with lead-containing materials, such patches can be applied to deeper tissues. Sun et al. [29] developed a wearable ultrasound blood-pressure monitoring patch using another lead-free material, sodium potassium niobate (KNN). The patch successfully measured changes in vascular diameter and established a relationship between blood pressure and vessel diameter, offering a safe, sustainable, comfortable, and wearable solution for long-term blood-pressure monitoring.

Beyond the commonly used piezoelectric materials described above, certain natural biomaterials and novel composites have also been incorporated into ultrasound patch designs to meet specific requirements such as biocompatibility and bioactivity. Chakraborty et al. [30] prepared chitosan (CHT) films via solvent-casting followed by cross-linking in an alkaline solution. Under ultrasonic stimulation, the CHT films exhibited notable antibacterial and anti-inflammatory activities, along with inhibition of inflammatory cytokines. Liu et al. [31] developed a bilayer-structured BTO@PCL/GO@GelMA nanopatch, in which a barium titanate-polycaprolactone (BTO@PCL) nanofiber membrane serves as the piezoelectric generation layer, and a graphene oxide-gelatin methacryloyl (GO@GelMA) hydrogel layer functions as the neural interface. Under low-intensity pulsed ultrasound (LIPUS) stimulation, the patch converts mechanical energy into electrical energy to deliver wireless electrical stimulation, significantly promoting peripheral nerve repair and functional recovery. This illustrates the integrated advantages of piezoelectric composites in neuromodulation and tissue regeneration.

The evolution of materials for ultrasound patches, as summarized in Table 1, clearly demonstrates a progression from prioritizing single functional attributes to pursuing a holistic balance of performance, flexibility, and biosafety. While the field has successfully moved from rigid, toxic lead-based ceramics towards flexible polymers and biocompatible lead-free alternatives, a critical trade-off persists: the lower piezoelectric output of these safer, more flexible materials often limits the sensitivity and penetration depth required for demanding deep-tissue applications. Current research addresses this through intricate composite systems (e.g., BTO@PCL/GO@GelMA), which, despite their design versatility, introduce significant complexity in fabrication and raise questions about long-term interfacial stability under dynamic in vivo conditions. This suggests that the next frontier in materials science for this field is not merely the discovery of a single “perfect” material, but the development of robust, scalable manufacturing processes for reliable, multi-material composites that can seamlessly integrate high performance with proven long-term biocompatibility.

## 3. Structure Design

### 3.1. Transducer Structural Innovation and Miniaturization

**Figure 1:**
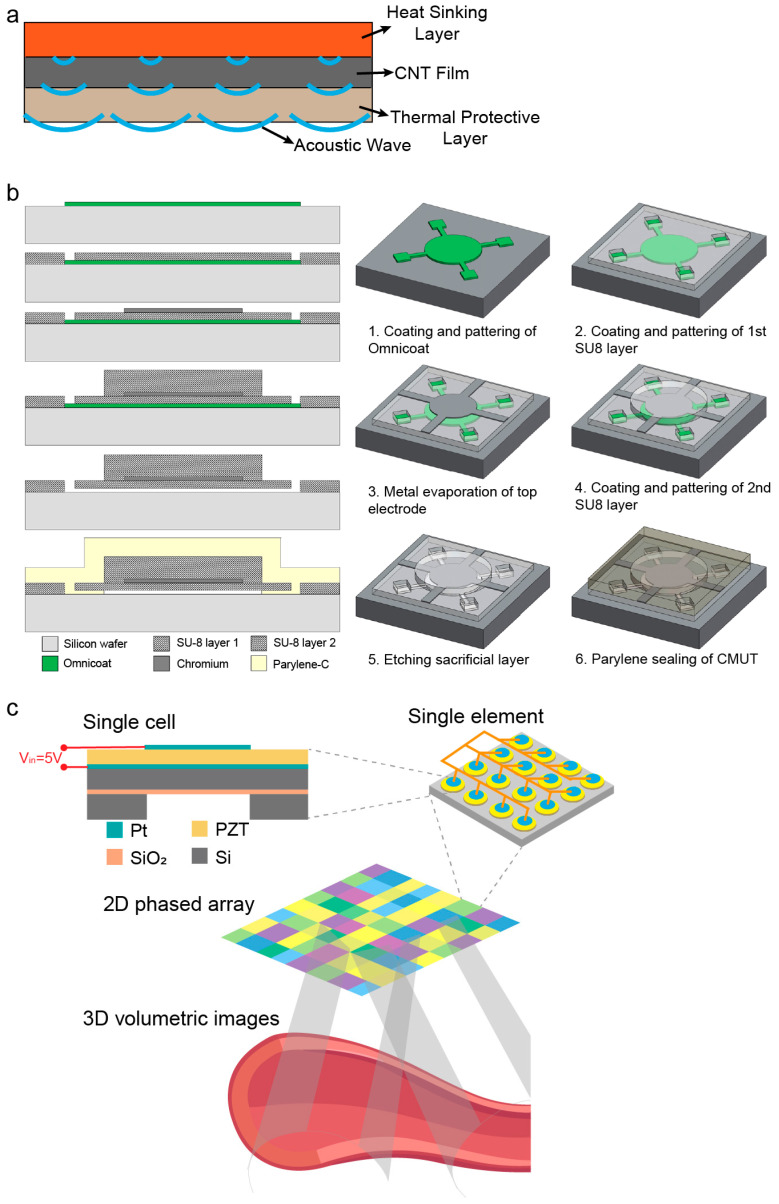
Advances in transducer structural innovation and miniaturization for ultrasound patches. (**a**) Schematic of a flexible ultrasound patch based on a carbon nanotube (CNT) film sandwich structure, consisting of piezoelectric units, a thermal protection layer, and a heat dissipation layer for conformal attachment to irregular surfaces. Inspired by Feng et al. [32]; (**b**) Fabrication process flow of polymer-based capacitive micromachined ultrasonic transducers (polyCMUTs), illustrating the use of SU-8 polymer and embedded electrodes to achieve a low-cost, low-voltage design. Reproduced from Gerardo et al. [33], under the CC BY 4.0 license; (**c**) Hierarchical “cell–element–array” architecture of a MEMS-based piezoelectric micromachined ultrasonic transducer (PMUT) phased array, driven by 5 V programmable pulses to enable beamforming and volumetric imaging. Inspired by Zhang et al. [35].

The enhancement of core performance in ultrasound patches primarily relies on breakthrough designs in both piezoelectric materials and transducer architecture. Traditional rigid piezoelectric ceramics, due to their poor conformity to human body contours, severely constrain wearing comfort and acoustic field stability. Consequently, flexible piezoelectric composites have emerged as a crucial solution. Feng et al. [32] developed a flexible ultrasound patch based on carbon nanotube (CNT) films. Employing a sandwich structure composed of a CNT film, a thermal protective layer, and a heat sinking layer (Figure 1a), the patch generates ultrasound via the thermoacoustic effect and achieves adaptive conformal attachment to irregular body surfaces. This design offers a promising foundation for lightweight, wearable therapeutic devices. Gerardo et al. [33] proposed a design for low-cost, polymer-based capacitive micromachined ultrasonic transducers (polyCMUTs). This approach substitutes traditional piezoelectric ceramics with the photosensitive polymer SU-8 and incorporates an embedded electrode design, featuring low operating voltage and high sensitivity, thereby significantly reducing energy consumption and fabrication complexity (Figure 1b). Inspired by traditional Chinese mortise-and-tenon joinery, Liu et al. [34] composited an amino-anchored metal–organic framework (MOF) with PVDF, resulting in a 40% increase in β-phase content, a 550% enhancement in remnant polarization, and improved X-ray responsiveness, laying a material foundation for integrated diagnosis and therapy.

Beyond material systems, the structural miniaturization of transducers via microelectromechanical systems (MEMS) processes represents another key pathway to reconcile the demands of deep-tissue imaging and implantable applications. Zhang et al. [35] developed a 2D PMUT array using MEMS technology, which innovatively adopts a three-level driving architecture of “unit-element-array” (Figure 1c). Operating at only 5 V, it achieves 3D volumetric imaging at a frame rate of 11 kHz (covering 40 × 40 × 70 mm^3^), paving the way for long-term wearable imaging of various organs within deep tissues. Gami et al. [36] demonstrated the feasibility of PMUTs for wearable vascular imaging, finding comparable performance in pulse wave imaging (PWI) between a miniaturized PMUT array and a clinical L7-4 probe, thereby opening new avenues for cardiovascular health monitoring.

### 3.2. Array Configuration and Acoustic Field Control

**Figure 2:**
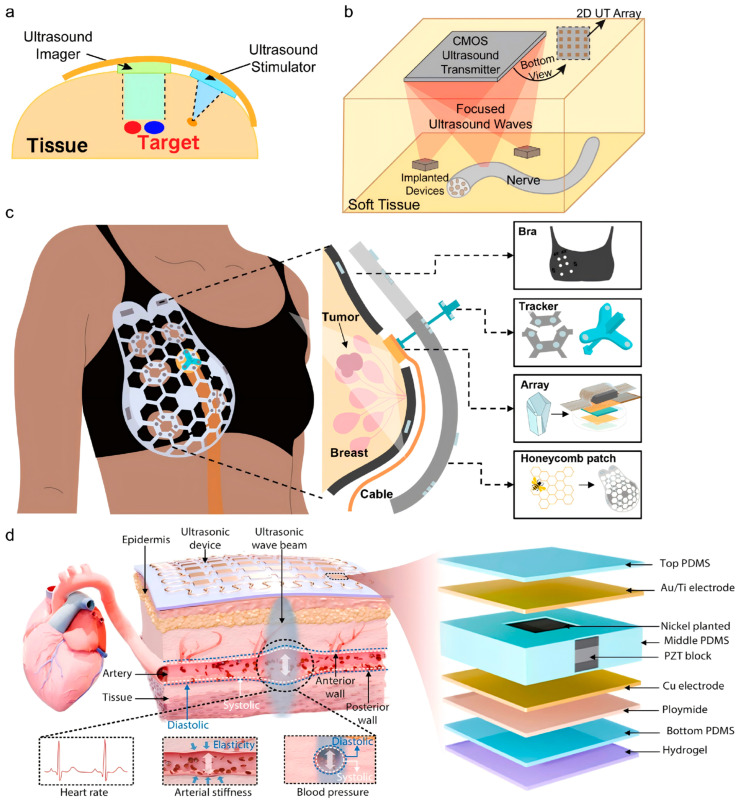
Innovative designs in ultrasound patch array configuration and acoustic field control for enhanced diagnostic and therapeutic precision. (**a**) A wearable dual-mode ultrasound probe wrapped around a body part, enabling integrated imaging and neuromodulation through flexible array design. Inspired by Huan et al., 2025 [38]; (**b**) Miniaturized ultrasound transducers integrated onto a CMOS chip, allowing pixel-matched beamforming and precise 3D focal-spot positioning for applications such as powering implantable devices and targeted neuromodulation. Inspired by Costa et al., 2021 [39]; (**c**) Exploded view of a honeycomb-structured conformable ultrasound breast patch that combines a soft fabric bra, a honeycomb guidance layer, a rotatable tracker, and a single-crystal 1D phased array to enable large-area, deep-tissue, multi-angle breast scanning. Reproduced from Du et al., 2023 [41]; (**d**) Schematic of a skin-adaptive focused ultrasound patch that uses the natural curvature of the skin as an acoustic lens to dynamically adjust beam width and depth for stable hemodynamic monitoring at highly curved vascular sites. Reproduced from Yuan et al., 2025 [42].

To accommodate complex anatomical structures, multi-modal integrated arrays have become key to enhancing diagnostic and therapeutic precision. Pashaei et al. [37] designed a body-conforming dual-mode patch integrating a 64-element, 5 MHz imaging array with an 8-element, 1.3 MHz neuromodulation array. By utilizing real-time strain-sensing feedback to optimize acoustic beam focusing and combining high-voltage multiplexing technology, they achieved a 5 dB improvement in echo signal-to-noise ratio, enabling precise targeting of structures like the vagus nerve. Similarly, Huan et al. [38] assembled imaging (4 MHz) and neuromodulation (1.3 MHz) transducers on a flexible printed circuit board (Figure 2a), pioneering a proportional-integral controller based on electromyographic feedback to dynamically adjust ultrasound intensity and compensate for inter-individual variability.

Regarding high-density array design, Costa et al. [39] proposed a pixel-matched beamforming technique, directly integrating a 26 × 26 piezoelectric ultrasound transducer array onto a complementary metal-oxide-semiconductor (CMOS) chip (4 × 5 mm^2^) (Figure 2b). Operating at 8.4 MHz with a 5 V supply, the device achieves focal pressures up to 100 kPa and focal spots of ~200 μm, enabling precise 3D positioning for powering implantable devices and neuromodulation. However, as the authors note, higher driving voltages or longer pulse durations would require careful monitoring of heat dissipation to ensure safe operation [39]. Thinning the CMOS substrate has been proposed as a pathway toward future wearable implementations. Chen et al. [40] innovatively developed a transparent ultrasonic transducer (TUT) array using lithium niobate crystals, enabling synchronous quad-modal imaging combining photoacoustic, ultrasound, Doppler, and fluorescence techniques. When the 64-element, 6 MHz array is directly coupled to tissue, it can resolve blood vessels and tumors with high resolution, offering a novel tool for endoscopic and wearable imaging.

For imaging highly curved organs, biomimetic structural designs significantly improve acoustic performance. Du et al. [41] developed a honeycomb-structured conformable ultrasound breast patch (Figure 2c). Mechanically, the honeycomb design combines a flexible TPU layer and a rigid PLA layer, enabling conformal attachment to curved breast surfaces and supporting 360° rotation of the attached 1D phased array via a magnetically guided tracker. Acoustically, this configuration allows multi-angle, deep-tissue scanning across up to 15 predefined positions, achieving a field of view comparable to commercial probes at depths > 30 mm. The patch thus enables standardized, operator-independent breast imaging with high repeatability. Yuan et al. [42] reported a skin-adaptive focused ultrasound patch (Figure 2d). Its array employs a biomimetic design that utilizes the skin’s own curvature as a natural acoustic lens, allowing the ultrasound beam width (2.1–4.6 mm) and depth (3.3–53.0 mm) to adaptively match the dimensions and locations of subcutaneous blood vessels under varying curvatures (radius: 10–60 mm). This achieves stable, high signal-to-noise ratio hemodynamic monitoring at sites with highly variable curvature, such as the radial and carotid arteries. These works demonstrate that biomimetic structural design effectively addresses the adaptation challenge for complex anatomical surfaces and is a key pathway for enhancing the performance of wearable ultrasound imaging.

### 3.3. Lightweight Structures and Biointerfaces

The long-term usability of wearable devices demands that structural design achieves lightweight characteristics while simultaneously ensuring biocompatibility and operational convenience. This is primarily manifested in three aspects: lightweight therapeutic architectures for surface-level conditions, intelligent structures enabling precise transdermal delivery, and tissue-interfacing designs that guarantee signal quality.

**Figure 3:**
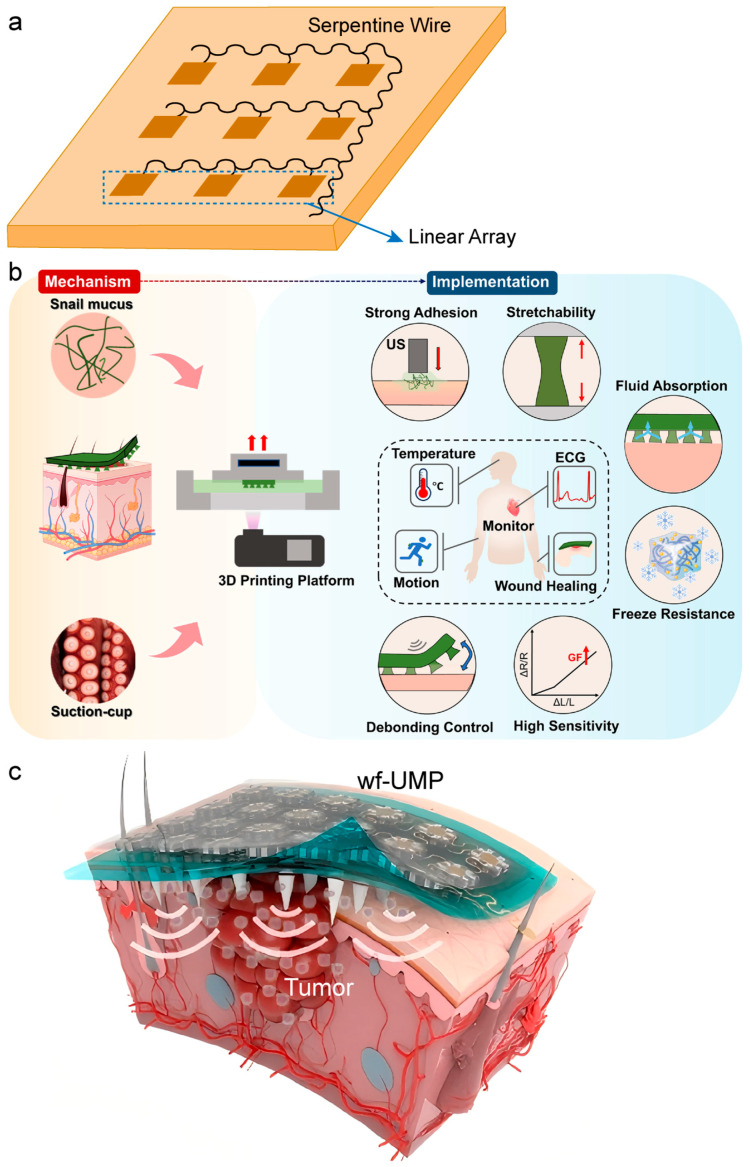
Structural and interfacial innovations for enhanced conformability and biointegration of ultrasound patches. (**a**) “Island-bridge” circuitry design employing discretized piezoelectric ceramic units interconnected by serpentine traces, enabling mechanical flexibility and stable electrical performance under deformation. Inspired by Lyu et al. [44]; (**b**) 3D-printed, ultrasound-mediated multi-coupled bioinspired adhesive hydrogel interface, mimicking sucker and mucus mechanisms to achieve spatiotemporally controllable, tough yet detachable adhesion for robust skin-electronics coupling. Reprinted from Ma et al., 2024 [46], with permission from Elsevier; (**c**) Schematic of a wearable flexible ultrasound microneedle patch for localized cancer therapy, integrating drug delivery with acoustic actuation in a single conformal platform. Adapted from Xue et al., 2025 [47], with permission from Springer Nature.

In the field of chronic wound therapy, Ngo et al. [43] employed a disk-shaped patch architecture (diameter 40 mm, weight < 20 g) that can be directly embedded into dressings. Applying non-thermal ultrasound (20–100 kHz, intensity 100 mW/cm^2^) for safe treatment durations of up to 4 h, this approach reduced diabetic ulcer healing time from 12 weeks to 4.7 weeks. Lyu et al. [44] constructed a conformable ultrasonic patch by discretizing the piezoelectric ceramic into a linear array of units integrated with flexible “island-bridge” circuitry and serpentine interconnects (Figure 3a). Owing to its unique bending-induced acoustic beam self-focusing capability, the patch achieved a reduction in wound healing time by approximately 40% in a type II diabetic rat model. For transdermal delivery scenarios, Huang et al. [45] embedded drug-loaded polyester microcapsules within a four-arm polyethylene glycol (PEG) hydrogel patch. Ultrasound was utilized to synchronously trigger drug release and enhance transdermal efficiency. In vitro experiments demonstrated negligible drug permeation in the absence of ultrasonic stimulation, achieving precise spatiotemporal control.

The adhesive stability of the biointerface directly impacts signal quality. Ma et al. [46] developed a multi-level coupled hydrogel interface (PAMS patch) by mimicking the biological structures of octopus suckers and snail mucus (Figure 3b), establishing a stable and intimate mechano-electronic coupling at the tissue-electronic interface. Xue et al. [47] constructed an integrated bioelectronic wearable platform by combining a stretchable lead-free ultrasound array, a bioadhesive hydrogel, and dissolvable microneedles (Figure 3c). The tight coupling between its flexible substrate and the tissue surface provides a robust foundation for sono-immunotherapy of tumors.

The structural innovations detailed here collectively address the fundamental challenge of conformably and efficiently coupling acoustic energy with the human body. The move towards MEMS-based arrays (e.g., PMUTs) and multi-modal integration has successfully enhanced imaging resolution and enabled closed-loop feedback, as seen in the strain-sensing neuromodulation patches [37,38]. However, this increasing structural complexity often comes at the cost of higher fabrication complexity and potential vulnerability. For instance, while biomimetic designs like the honeycomb breast patch or skin-adaptive lens elegantly solve the problem of adapting to complex curvatures, their performance is predicated on a specific, idealized deformation. A critical, unanswered question is how these intricate structures will behave under the unpredictable, multi-axial strains and deformations encountered during daily human activity over extended periods. The true test for these designs lies not just in their ex vivo precision, but in their long-term mechanical and acoustic reliability in the messy, dynamic reality of the human body.

## 4. Application Scenarios

**Figure 4:**
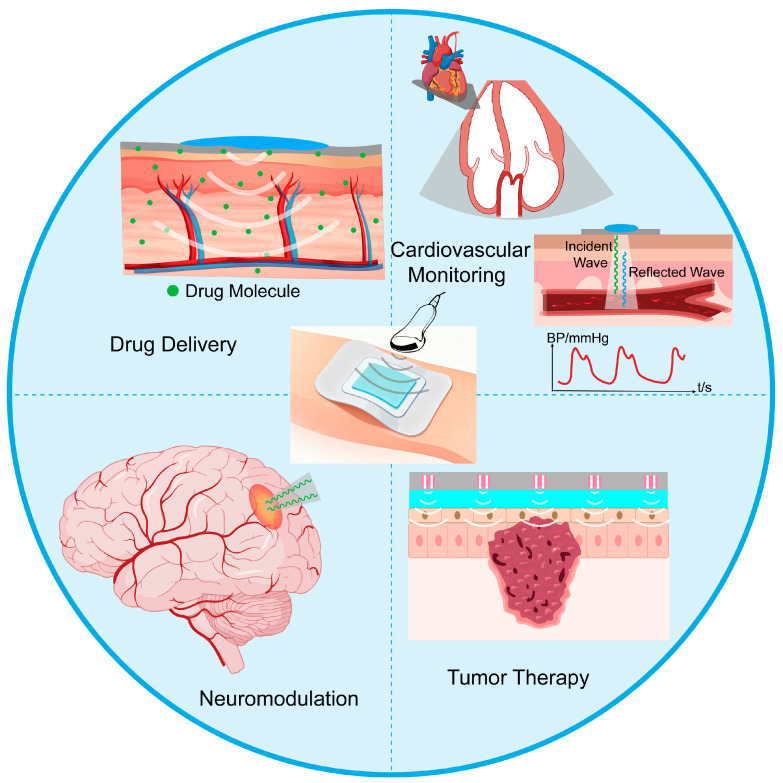
Schematic diagram illustrating the application of ultrasonic patches in various scenarios.

Building upon innovations in materials and transducer architectures, ultrasound patches are unlocking a spectrum of closed-loop biomedical applications (Figure 4). Compared to traditional rigid ultrasound probes, ultrasound patches represent a breakthrough innovation in terms of wearability, tissue adaptability, and functional integration. Conventional probes are limited by their rigid substrates and dependence on liquid coupling agents, making it difficult to achieve conformal contact with irregular body surfaces, which compromises signal stability and may increase the risk of wound infection [44]. In contrast, flexible patches, through the miniaturization of piezoelectric materials [39] and integration with elastic substrates [10], can seamlessly conform to skin curvatures, significantly improving detection accuracy and therapeutic reliability. Furthermore, the patch-based design facilitates long-term continuous monitoring, providing dynamic data support for chronic disease management (e.g., cardiovascular diseases, diabetes) [7,48]. This “unobtrusive wearability” characteristic greatly enhances patient compliance and creates conditions for home-based health management [49].

### 4.1. Disease Diagnosis and Imaging

Innovations in ultrasound patches within the field of diagnostic imaging are first exemplified by their ability to enable long-term, continuous monitoring of deep-seated tissues such as those in the cardiovascular system. Compared to traditional bulky ultrasound equipment, the flexible patch design effectively overcomes signal attenuation and probe displacement caused by patient movement, significantly enhancing signal stability and data reliability during dynamic monitoring. Their superior performance has been validated in several pioneering studies: Lin et al. [7] developed a fully integrated, autonomous wearable USoP system. By incorporating miniaturized flexible control circuits and machine learning algorithms, the system achieves automatic tracking of moving targets (e.g., a beating heart) and can continuously monitor physiological signals from tissues up to 164 mm deep—such as central arterial blood pressure, heart rate, and cardiac output—for up to 12 h. This system represents a significant breakthrough in wearable ultrasound diagnostics toward prolonged, dynamic deep-tissue monitoring, offering a novel tool for at-home cardiovascular health management. Furthermore, the patch design has also been applied to emerging imaging modalities. For example, the photoacoustic patch developed by Gao et al. [50] achieved, for the first time, 3D imaging of hemoglobin at subcutaneous depths > 2 cm, demonstrating the platform’s substantial potential in expanding the detection depth and informational dimensions of wearable imaging.

Enhancements in imaging performance rely on synergistic innovations in piezoelectric material systems and miniaturized devices. Regarding lead-free piezoelectric materials, the wearable ultrasound patch based on KNN developed by Sun et al. [29] employs biocompatible silicone rubber encapsulation, enabling tight skin conformal attachment without the need for coupling gel. By measuring changes in vascular diameter and establishing a quantitative relationship with blood pressure, its reliability for continuous non-invasive blood pressure monitoring was validated in an in vitro simulation system. In terms of device architecture, the silicon nanopillar capacitive micromachined ultrasonic transducer (snCMUT) array by Kang et al. [10] combines lead-free design and miniaturized fabrication (thickness ~900 μm) with high transmission efficiency, excellent flexibility, and low power consumption. Operating at low voltage, this device achieves high resolution and a penetration depth of approximately 70 mm, and has been successfully applied to high-definition imaging of the human carotid artery and continuous blood pressure monitoring, fully demonstrating the pivotal role of miniaturization in improving wearability and imaging quality.

The functionality of ultrasound patches is expanding from pure imaging toward integrated systems featuring multimodal sensing and image-guided capabilities, laying the foundation for their application in theranostics. In multimodal sensing, Sempionatto et al. [48] developed an epidermal patch that innovatively integrates an ultrasound transducer with electrochemical sensors on a single flexible platform. To prevent crosstalk, the acoustic and electrochemical components were spatially separated by 1 cm and isolated using solid-state hydrogel layers. This design enables reliable simultaneous monitoring of hemodynamic parameters (blood pressure, heart rate) and metabolic biomarkers (glucose, lactate, alcohol, caffeine) without signal interference, as validated through on-off switching tests. In image guidance, the body-conforming dual-mode ultrasound patch for image-guided neuromodulation designed by Pashaei et al. [37] introduced an algorithm that uses adjacent blood vessels (e.g., the carotid artery) as imaging landmarks for automatic target nerve localization, establishing a basis for precise neural therapies.

### 4.2. Drug Delivery

Ultrasound patches revolutionize drug delivery and neuromodulation strategies through synergistic energy-matter delivery mechanisms. In the field of transdermal administration, these patches integrate physical permeation-enhancing techniques with intelligent drug release technologies, significantly improving the delivery efficiency of biological macromolecules.

Regarding the fusion of nanocarriers with patches, researchers are dedicated to embedding drug-loaded nanoparticles within biocompatible patch matrices, combined with ultrasound-controlled release, to achieve efficient and sustained drug delivery. Ali et al. [51] developed a chitosan patch loaded with repaglinide solid lipid nanoparticles (REP-SLN-TDDS). This patch successfully dispersed SLNs uniformly within its matrix and exhibited biphasic release characteristics: approximately 36% of the drug was released within the first 2 h, with cumulative release reaching 80% over 24 h. Most importantly, its transdermal flux was enhanced by 3.56-fold. In vivo studies in rats demonstrated that it could maintain a more prolonged and effective plasma drug concentration and significantly lower blood glucose levels, proving its potential for improving the delivery of anti-diabetic drugs with low oral bioavailability. Similarly, Li et al. [52] prepared chitosan-alendronate sodium nanoparticles for osteoporosis treatment, loaded them into a hydroxypropyl methylcellulose patch, and combined them with ultrasound-enhanced permeation. This approach increased the bioavailability by 6-fold compared to a conventional patch in rats and was more effective in reducing serum calcium levels (from 16 mg/dL to 4 mg/dL), highlighting the advantage of nanoparticle-ultrasound patch combinations in promoting the transdermal delivery of macromolecules and poorly soluble drugs.

Microstructured patch designs, combined with ultrasound and other physical enhancement strategies, further overcome skin barrier limitations by actively creating microchannels or leveraging synergistic physical effects. This is particularly suitable for macromolecule delivery or localized therapy. Bok et al. [53] designed a unique needle-free microcup patch integrating a drug reservoir (loaded with an ultra-thin salmon DNA-based drug film), an adhesion system, and physical stimuli sources (ultrasound, electric current). In the presence of moisture, the DNA film dissolves to release the drug. Combined with ultrasound or electrical stimulation, it significantly promotes drug penetration through the stratum corneum and the entire epidermis while reducing skin irritation. Another study [54] developed a multifunctional system composed of hyaluronic acid microneedles, where the drug load at the needle tips was controlled by adjusting solution concentration. It was found that ultrasonic cavitation pressure vibrations induced microneedle dissolution, while alternating current iontophoresis enhanced the electro-osmotic driven diffusion of charged molecules (e.g., Rhodamine B). This synergistic effect of ultrasound and iontophoresis within the microneedles significantly shortened the initial delivery time and increased permeability compared to passive diffusion or ultrasound alone, offering a new approach for macromolecular drugs and time-dependent delivery. Li et al. [55] constructed a Piezoelectric-driven Microneedle Array (PDMA) for psoriasis treatment. A finite element model confirmed that PDMA could generate an ultrasonic field. In vitro experiments showed PDMA increased the penetration depth of methotrexate (MTX) by 9-fold. In vivo studies demonstrated that PDMA-mediated MTX delivery was significantly superior to oral administration in alleviating psoriasis symptoms, achieving better efficacy with only 50% of the oral dose, providing an efficient and minimally invasive alternative for localized treatment.

Intelligent stimuli-responsive hydrogel and microcapsule patches focus on achieving on-demand and precise drug delivery, leveraging materials responsive to external stimuli (primarily ultrasound) for controlled therapy. Huang et al. [45] developed a hydrogel patch by embedding diclofenac sodium (DS)-loaded polyester microcapsules into a four-arm PEG matrix. Ultrasound exposure triggered rapid drug release from the microcapsules and simultaneously enhanced skin permeation, while negligible release occurred without stimulation—offering potential for on-demand treatment of arthritis and soft tissue injuries. Zhang et al. [56] designed a sonoelectric patch combining a piezoelectric PVDF film with micron-sized gas cavitation bubbles. Under ultrasound, the bubbles converted acoustic energy into electricity, generating a cross-film voltage of ~90 mV; the synergistic effect of ultrasonic pressure and the electric field boosted transdermal delivery, achieving 100% higher plasma concentration than ultrasound alone in animal studies. Beyond these chemically crosslinked or physically structured systems, advanced fabrication techniques such as 3D microextrusion printing have recently enabled the development of hydrophilic silicone-based hydrogels with precisely tailored drug release kinetics and excellent biocompatibility [57]. These materials exhibit high elasticity (elastic modulus ~15 kPa), negligible permanent deformation, and sustained release profiles (~20% cumulative release over 8 h), offering a complementary platform for ultrasound-mediated drug delivery when integrated with conformable patches.

Comparative studies on ultrasound parameter optimization and physical permeation enhancement technologies provide crucial evidence for clinical application. Vaidya et al. [58] compared the effects of three physical permeation enhancement techniques (ultrasound, electroporation, cold laser) on the transdermal efficacy of an MTX patch for treating rheumatoid arthritis (RA). In vitro studies indicated that ultrasound (via a sonoporation effect) offered the best permeation enhancement. Pharmacodynamic experiments in a rat RA model confirmed that the group receiving the MTX patch following ultrasound pretreatment showed significantly greater reduction in hind paw swelling, improved mobility scores, and pain relief compared to the group using only the MTX patch, with animals recovering faster. This established the value of ultrasound as an efficient permeation enhancer for MTX patches in RA treatment.

### 4.3. Neuromodulation

The application of ultrasound patch technology in the field of neuromodulation is gradually revealing its unique potential for non-invasive intervention, which hinges on the precise modulation of ion channel activity and neural network excitability through mechanical and thermal effects. A number of studies utilizing patch-clamp techniques have delved into the molecular mechanisms by which ultrasound influences neuronal electrical activity. Cui et al. [59] found that ultrasound stimulation could significantly suppress voltage-gated potassium currents (including transient outward and delayed rectifier potassium currents) in rat hippocampal CA1 pyramidal neurons. This inhibition of potassium efflux directly led to an increase in the frequency of spontaneous neuronal firing. This phenomenon was further explored mechanistically by Prieto et al. [60], who reported a frequency-dependent bidirectional modulation of action potential firing in CA1 neurons by high-frequency ultrasound (43 MHz): ultrasound suppressed action potentials when neurons received low input currents (near threshold) and fired at low frequencies, but enhanced firing frequency under conditions of high input current and high firing frequency. The researchers proposed that ultrasound might achieve this modulation by activating a standing potassium conductance, potentially via thermally or mechanically sensitive two-pore domain potassium (K2P) channels, with finite element modeling suggesting a temperature rise of less than 2 °C as the primary factor. Sorum et al. [61,62], through quantitative membrane tension measurements and fluorescence imaging, confirmed the high sensitivity and broad response range of mechanosensitive K2P channels to tension, and demonstrated that low-intensity, low-frequency focused ultrasound could activate these channels by increasing membrane tension, providing experimental evidence for the direct action of ultrasound-mediated mechanical force on neuronal membranes.

At the level of calcium signaling and synaptic transmission, ultrasound stimulation demonstrates a complex capacity to regulate neural network activity. Fan et al. [63] demonstrated that low-intensity pulsed ultrasound (LIPUS) significantly enhanced the frequency of spontaneous action potentials, as well as the frequency and amplitude of excitatory postsynaptic spontaneous currents (EPSCs) in cultured hippocampal neurons, with effects persisting for over 10 min post-stimulation. Combining calcium imaging, they elucidated that LIPUS promotes an increase in cytosolic calcium concentration via L-type calcium channels (LTCCs), subsequently activating the CaMKII-CREB pathway to regulate gene transcription. Li et al. [64] further observed that LIPUS evoked significant increases in both the frequency and amplitude of EPSCs in high-density cultured hippocampal neurons, indicating enhanced glutamatergic synaptic transmission. Mechanistic analysis revealed that extracellular calcium influx, action potential firing, and synaptic transmission were necessary for this response. Concurrent calcium imaging showed that LIPUS could recruit recurrent excitatory network activity in high-density cultures, lasting for tens to hundreds of seconds, highlighting the potent regulatory potential of ultrasound on neural network cascades.

Research on the application of ultrasound in treating neurological disorders is advancing from animal models toward clinical translation, with notable progress particularly in epilepsy intervention. Lin et al. [65] applied LIPUS (750 kHz, 0.35 MPa) to stimulate the epileptogenic focus for 30 min in a penicillin-induced epilepsy macaque model. This treatment significantly reduced both the total number of seizures (sham group: 107.7 ± 1.2; ultrasound group: 66.0 ± 7.9) and the hourly seizure frequency (sham group: 15.6 ± 1.2; ultrasound group: 9.6 ± 1.5) over a 16 h period. In ex vivo experiments on human epileptic brain slices, 28 MHz ultrasound (0.13 MPa) suppressed over 65% of epileptiform activity. Xu et al. [66] innovatively combined sonogenetics technology, specifically expressing the mechanosensitive ion channel MscL-G22S in parvalbumin (PV)-positive and somatostatin (SST)-positive inhibitory interneurons in the hippocampal CA1 region, followed by ultrasound stimulation. Results showed that activation of PV interneurons induced by MscL-G22S-mediated sonogenetics (MG-SOG) effectively ameliorated kainic acid (KA)-induced status epilepticus (SE) in mice and corrected SE-associated electrophysiological abnormalities in the CA1 region, while activating SST interneurons was ineffective. This provides a novel strategy for precise ultrasound modulation targeting specific neural circuits. Furthermore, Zou et al. [67] developed a portable, integrated wearable ultrasound system. Using a flexible honeycomb-structured ultrasound array patch, they achieved continuous treatment in a mouse model of familial Alzheimer’s disease (FAD). The system effectively reduced cerebral β-amyloid (Aβ) deposition, improved cognitive function, and promoted microglial phagocytosis of Aβ plaques and their polarization toward an anti-inflammatory M2 phenotype, opening a new avenue for the non-invasive treatment of neurodegenerative diseases.

In summary, ultrasound modulates neural function through multi-scale mechanisms (ion channels, synaptic transmission, network activity) and demonstrates broad clinical application prospects in intervening in major neurological disorders such as epilepsy and Alzheimer’s disease. From the elucidation of fundamental ion channel mechanisms to the development of precise sonogenetic modulation strategies, and onward to the clinical translation of wearable devices, this field is advancing toward a new era of efficient, precise, and personalized neuromodulation.

### 4.4. Tumor Diagnosis and Therapy

As an emerging platform for transdermal drug delivery and therapy, ultrasound patches are driving innovation in oncology toward integrated and precise theranostic models. By integrating technologies such as sonosensitive materials, piezoelectric components, and microneedles, they enable precise tumor monitoring, drug delivery, and synergistic therapies, significantly improving treatment efficacy and safety.

The key to advancing tumor theranostics with ultrasound patches lies in their ability to integrate real-time monitoring with immediate therapeutic intervention on a single platform, forming a dynamic management loop. This concept transcends mere diagnosis or treatment, aiming to optimize therapeutic processes through real-time feedback. For instance, Siboro et al. [68] developed a thermoplastic polyurethane (TPU) film patch based on hafnium oxide nanoparticles (HfO_2_ NPs). This patch functions as a dielectric elastomer strain sensor, monitoring impedance changes induced by tumor volume variation in real time. It ingeniously combines diagnostic and therapeutic functions: as a dielectric elastomer strain sensor, it can wirelessly assess disease progression by detecting impedance changes from tumor volume; simultaneously, the loaded HfO2 NPs act as sonosensitizers, generating reactive oxygen species (ROS) under ultrasound irradiation to directly kill cancer cells. This “monitoring-therapy” integrated design provides a highly promising tool for achieving personalized, dynamic tumor treatment.

Regarding specific therapeutic strategies, the ultrasound patch platform primarily supports two major innovative modalities: active drug delivery and in situ activation therapy. Active drug delivery strategies focus on utilizing the patch to physically breach biological barriers and precisely transport therapeutic agents to the tumor site. Xue et al. [47] proposed an integrated wearable flexible ultrasound microneedle patch (wf-UMP), which combines a stretchable lead-free ultrasound transducer array, a bioadhesive hydrogel, and drug-loaded dissolvable microneedles. It efficiently delivers anticancer drugs, not only inducing tumor cell apoptosis but also, when combined with immune checkpoint inhibitors, activating systemic anti-tumor immunity and inhibiting distant metastasis. The in situ activation therapy strategy relies on the patch generating a controllable ultrasound field to activate pre-accumulated or intrinsic sonosensitive substances at the tumor site, producing therapeutic effects locally without the need for complex delivery systems. Zou et al. [16] developed a fully integrated conformal wearable ultrasound patch (CWUS Patch). Through a multi-channel ultrasound array that precisely focuses on the lesion area, it controllably activates sonosensitizers to generate abundant ROS, enabling continuous sonodynamic therapy. This study validated the ability of ultrasound to penetrate deep tumor tissues in a mouse breast cancer model, demonstrating its potential for non-invasive, continuous, and efficient treatment of deep-seated tumors. Currently, many advanced nano-formulations [69,70,71] designed to enhance sonodynamic efficacy also heavily depend on a portable platform capable of providing localized, controllable ultrasound fields. This further underscores the pivotal role of ultrasound patches as a crucial hub in achieving precise and minimally invasive tumor therapy.

### 4.5. Other Applications

Beyond the core diagnostic and therapeutic domains discussed above, ultrasound patches are rapidly expanding into broader scenarios of health monitoring and rehabilitation intervention, demonstrating particular value in chronic disease management and functional recovery.

In the field of urological management, real-time monitoring for patients with neurogenic bladder dysfunction represents a typical application. Multiple studies have validated the feasibility of wearable ultrasound patches in this context. For instance, Cai et al. [12] utilized a commercial wearable device to achieve real-time bladder volume monitoring in pediatric patients. Cao et al. [72] and Pu et al. [73] developed an acoustic focusing system integrated with machine learning algorithms and a stretchable transducer array, respectively, enabling accurate, non-invasive bladder volume estimation while addressing the challenge of conformal attachment to the skin. In a pilot clinical study, Zhang et al. [74] demonstrated that their conformal ultrasound patch based on multiple phased arrays achieved bladder volume estimation errors comparable to standard clinical equipment, with the advantage of simpler operation.

In the assessment and rehabilitation of the musculoskeletal system, ultrasound patches can monitor dynamic changes in muscle morphology and mechanical properties in real time, providing objective quantitative feedback for rehabilitation training. Tang et al. [75] employed a miniaturized wearable ultrasound system with pulsed-wave Doppler imaging to monitor muscle contractions, revealing that waveform patterns and velocity can reflect an individual’s muscle function status. The distributed ultrasound sensing system developed by King et al. [76], combined with machine learning, successfully predicted ground reaction force during isometric exercises. At the therapeutic level, Cao et al. [77] integrated wearable ultrasound with functional electrical stimulation (FES). By using ultrasound to accurately identify stroke patients’ movement intentions, they facilitated the improvement of wrist function, thereby constructing an active closed-loop rehabilitation system.

Preliminary progress has also been made in the continuous monitoring of other critical physiological signals. For example, Wang et al. [78] reported the first application of a wearable ultrasound device for continuous, non-invasive bedside cardiac monitoring in a neonate during perioperative management. Furthermore, Currens et al. [79] non-invasively detected lymphatic bubbles using ultrasound in a porcine model, offering a new approach for developing wearable devices to monitor decompression sickness risk. As monitoring capabilities continue to mature, ultrasound patches are evolving from pure diagnostic tools toward platforms integrating therapeutic functions. For instance, studies by Yang et al. [80] and Luo et al. [81] demonstrated the application of wearable ultrasound in intervening in type II diabetes and achieving dynamic blood glucose regulation, respectively, offering an initial glimpse into their potential as intelligent theranostic platforms that integrate sensing, decision-making, and intervention.

In summary, these studies collectively illustrate the broad prospects of ultrasound patches beyond traditional diagnostic imaging, gradually transforming from simple sensing platforms into proactive, real-time health management tools capable of intervention in home and rehabilitation settings.

To provide a comprehensive overview and facilitate direct comparison of the diverse ultrasound patch systems discussed in this review, we summarize the key characteristics of representative studies in Table 2. This comparative snapshot reveals several critical insights into the current state of the field. First, a clear divergence in material and structural priorities emerges across application domains: diagnostic imaging patches (e.g., snCMUT [10]) prioritize resolution and penetration via sophisticated MEMS-based arrays or high-performance piezoelectrics, while therapeutic patches (e.g., PDMA [55]) emphasize biocompatibility and interfacial integration, favoring flexible polymers, lead-free ceramics, and hybrid structures like microneedles or hydrogels. Second, the table highlights significant heterogeneity in reporting standards—key parameters for clinical translation, such as long-term biocompatibility or precise power consumption, are often inconsistently reported, underscoring the field’s nascent stage of development. Finally, and most importantly, while individual components of a closed-loop system—sensing (e.g., multimodal patch [48]), actuation (e.g., CWUS Patch [16]), and intelligent control—are being demonstrated in isolation, fully integrated platforms that autonomously combine these functions remain rare. The grand challenge of seamlessly unifying real-time diagnosis, on-device decision-making, and targeted therapy into a single wearable system is yet to be realized, and this transition from component-level innovation to system-level integration will define the next era of intelligent ultrasound theranostics.

## 5. Conclusions and Future Directions

A unifying vision emerging from the advances reviewed above is the evolution of ultrasound patches toward closed-loop intelligent theranostic systems. In this paradigm, real-time sensing informs adaptive therapeutic intervention, creating a dynamic and personalized treatment loop. Such a system comprises three core modules operating in concert: a sensing module continuously monitoring physiological parameters (e.g., hemodynamic signals [7], metabolic biomarkers [48], or neural activity [37]); an intelligent decision-making module, powered by machine learning algorithms, analyzing data to detect anomalies and determine optimal responses; and an actuation module executing targeted interventions such as focused ultrasound neuromodulation [38], controlled drug release [45], or sonodynamic therapy [16]. The resulting physiological changes are then sensed again, closing the loop and enabling continuous self-optimization. While individual components of this closed loop are being actively demonstrated (Table 2), truly autonomous systems that seamlessly unify sensing, on-device intelligence, and therapy without human intervention remain unrealized.

Realizing this vision requires overcoming persistent challenges across multiple fronts. Materials and device performance remain a fundamental bottleneck: the trade-off between the high piezoelectric output of lead-based ceramics and the flexibility and biosafety of lead-free alternatives persists, while intricate composite systems introduce concerns about long-term interfacial stability under dynamic in vivo conditions. Energy efficiency and power management pose another critical hurdle—low energy transfer efficiency due to beam steering errors [82] limits the practicality of ultrasound-powered implants, and achieving sufficient penetration depth (>10 cm) for deep-tissue applications while maintaining low power consumption for continuous wearability demands innovative circuit and energy harvesting strategies. Regulatory pathways and safety standardization are equally pressing: the wide variability in ultrasound parameters (frequency, intensity, duty cycle) across studies, coupled with the lack of established safety thresholds for long-term human exposure, hinders clinical translation. Furthermore, most current research remains confined to animal models or ex vivo experiments, with limited long-term human safety and efficacy data [49]. Finally, long-term wearability and reliability—including interfacial adhesion under dynamic motion, resistance to sweat and bodily fluids, and mechanical robustness under repeated deformation—must be rigorously validated before these patches can transition from laboratory prototypes to home-based clinical tools.

Several recent studies have demonstrated preliminary closed-loop architectures that integrate sensing, feedback, and therapy [7,37,38,48]. However, evidence on their safety, reversibility, and long-term metabolic effects remains limited. For instance, while Huan et al. [38] validated adaptive feedback control in an in vitro phantom, in vivo safety and reversibility data are lacking. Lin et al. [7] achieved 12 h continuous monitoring with machine learning-based target tracking, but the study did not assess the long-term effects of sustained ultrasound exposure on tissue health. Sempionatto et al. [48] demonstrated robust mechanical stability and no signal crosstalk during short-term human trials, yet the reversibility of metabolic modulation and potential cumulative effects remain unexplored. These gaps underscore the need for future closed-loop designs to systematically evaluate long-term safety, reversibility, and metabolic impact through well-controlled longitudinal studies.

Looking forward, the convergence of flexible hybrid electronics, low-power edge artificial intelligence (AI), and advanced energy harvesting will catalyze the emergence of fully integrated closed-loop ultrasound patches. In parallel, innovations in miniaturized and flexible antenna design are enabling seamless wireless data transmission and power delivery for body-area networks [83], further supporting the transition toward wireless, remote health monitoring. Future efforts should focus on three key areas: elucidating biological mechanisms (e.g., molecular targets for neuromodulation), conducting multicenter randomized controlled trials to validate long-term safety and efficacy in chronic disease management [80], neuromodulation [66,67], and tumor therapy [16], and establishing unified safety standards for ultrasound parameters. By transitioning from component-level innovation to system-level integration, ultrasound patches will ultimately realize their potential as intelligent platforms for personalized medicine, telemedicine, and home-based health management.

## Figures and Tables

**Table 1:** Comparison of key materials used in ultrasound patches.

Material Type	Examples	Advantages	Disadvantages
Piezoelectric Polymers	Polyvinylidene fluoride (PVDF) [24,25]	High flexibility and conformability, lightweight, good biocompatibility, simple and low-cost fabrication process.	Lower piezoelectric output compared to ceramics, which may limit sensitivity for some deep-tissue applications.
Lead-Containing Piezoelectric Ceramics	Lead zirconate titanate (PZT) [26] PMN-PZT [27]	Exceptionally high piezoelectric coefficients and electromechanical coupling, enabling high-sensitivity imaging and actuation.	Inherent rigidity requires composite design for wearability; contains toxic lead, raising biosafety and environmental concerns.
Lead-Free Piezoelectric Ceramics	Barium titanate (BTO) [28] Potassium sodium niobate (KNN) [29]	Excellent biosafety and environmental friendliness due to lead-free composition, with moderate to good piezoelectric performance.	Piezoelectric properties generally inferior to PZT; fabrication can be more challenging to achieve comparable performance.
Natural Biomaterials	Chitosan (CHT) [30]	Inherent biocompatibility, biodegradability, and bioactivity (e.g., antibacterial, anti-inflammatory), suitable for bioactive interfaces.	Mechanically weak and hydrolytically unstable unless chemically crosslinked; piezoelectric response is typically weak.
Piezoelectric Composites	BTO@PCL/GO@GelMA [31]	Design versatility to tailor flexibility, piezoelectricity, and additional functionalities (e.g., conductivity, bioadhesion) in a single system.	Complex fabrication; long-term reliability of the material interface under dynamic conditions needs further validation.

**Table 2:** Summary and comparison of representative ultrasound patch systems.

Application Scenarios	Study	Material/Structure	Dimensions/Thickness	Target/Function	Power/Voltage	Biocompatibility/Wearability Features	Working Principle	Intended Application
Disease Diagnosis and Imaging	USoP (Lin et al., 2024) [7]	Flexible US transducer array + integrated control circuit	Not specified	Deep tissue (up to 164 mm) physiological signals	a power consumption of ~614 mW	Fully integrated, wearable, continuous 12 h operation, motion tracking	Ultrasound imaging + machine learning	Continuous deep-tissue monitoring in moving subjects
	snCMUT (Kang et al., 2025) [10]	Silicon nanopillar CMUT array, flexible packaging	Overall thickness < 1 mm	Carotid artery imaging, BP waveform	8.9 Vpp (operating voltage)	Flexible, stretchable (except FPCB), disposable, no ASIC needed	Capacitive micromachined ultrasound transduction	Wearable cardiovascular monitoring patch
	BP Monitoring Patch (Sun et al., 2025) [29]	Lead-free piezoelect. (KNN-Cr), silicone rubber packaging	2.8 mm × 2.8 mm, center freq. 5 MHz	Radial artery, blood pressure detection	Not specified	Flexible, wearable, biocompatible, environmentally friendly	US measurement of vessel diameter change	Non-invasive continuous BP monitoring
	Multimodal Patch (Sempionatto et al., 2021) [48]	Integrated US transducer & electrochemical sensors	Not specified	BP, HR, and multiple biomarkers (glucose, lactate, etc.)	Not specified	Flexible epidermal patch, anti-crosstalk design, iontophoresis sampling	US monitoring + electrochemical sensing	Multimodal physiological & biochemical monitoring
	Photoacoustic Patch (Gao et al., 2022) [50]	VCSEL diode array + piezoelectric transducer	2.0 cm × 1.6 cm overall footprint, thickness 1.2 mm	Deep tissue hemoglobin imaging & core temperature	Not specified	Wearable, first continuous deep-tissue biomolecule monitoring	Photoacoustic imaging & thermometry	3D tissue imaging & core temperature measurement
Drug Delivery	Ultrasound-Responsive TDDS (Huang et al., 2019) [45]	PEG-PLGA microcapsules embedded in 4-arm-PEG hydrogel	Microcapsule diam. ~3.5 μm; patch diameter 10 mm; height 2 mm (for in vitro test)	Skin, transdermal drug delivery	2 W/cm^2^ (ultrasound intensity)	Good biocompatibility, excellent skin adhesion	Ultrasound-triggered drug release & permeation enhancement	Controlled transdermal delivery (e.g., arthritis)
	Transdermal REP-SLN (Ali et al., 2024) [51]	Chitosan-based transdermal system (Solid Lipid Nanoparticles)	Nanoparticle size ~249 nm	Skin, repaglinide delivery	Not specified	Transdermal patch, enhanced bioavailability	SLN prepared by ultrasound melt-emulsification	Transdermal delivery of antidiabetic drug
	PDMA (Li et al., 2025) [55]	PDMA (piezoelectric ceramic PZT8 + 3D-printed hollow microneedle array)	Device: φ44 mm × 26 mm; Needle: inner diameter 120 μm, outer diameter 180 μm, 1 mm height (3 × 3 array)	MTX, psoriasis treatment	200 Vpp, ~120 kHz, 0.56 W/cm^2^	Minimally invasive, no skin irritation, safe temp rise (5 °C)	Ultrasound cavitation-enhanced penetration (sonophoresis + microneedles)	Psoriasis therapy (9× penetration, 50% oral dose with better efficacy)
	Energy-Converting TDDS (Zhang et al., 2024) [56]	Microbubble & piezoelectric soft structure composite patch	PVDF film thickness 200 μm; patch size 2 × 2 cm	Skin, transdermal drug delivery	Not specified	Thermochromic indicator, safe operating temperature	US energy to electricity conversion, multi-field synergy for permeation	Enhanced & controlled transdermal delivery (e.g., pain)
Neuromodulation	Neuro-Modulation Patch (Pashaei et al., 2020) [37]	64-element imaging array + 8-element modulation array	Imaging: 0.4 mm thick; Modulation: 1.5 mm thick	Nerve localization & modulation (e.g., vagus)	Sensitivity: ~80 kPa/V (modulation array)	Body-conformal, integrated strain sensor for closed-loop feedback	Image-guided focused ultrasound neuromodulation	Neuromodulation therapy
	Epilepsy Therapy (LIPUS) (Lin et al., 2020) [65]	Conventional US transducer (non-patch form)	Not specified	Brain, neural excitability modulation	Low-intensity pulsed US	Non-patch form, investigational application	Low-intensity US neuromodulation	Epilepsy treatment
	Sonogenetics (MG-SOG) (Xu et al., 2024) [66]	Sonogenetics technique (non-device description)	Not specified	Hippocampal CA1 PV interneurons	650 kHz, 0.38 MPa	Research technique, non-wearable device	Sonogenetic control of specific neurons	Research on status epilepticus treatment
	AD Therapy Patch (Zou et al., 2025) [67]	Flexible honeycomb US array patch + flexible circuit	2 mm thick	Brain, amyloid-β plaque disaggregation	1 MHz, 1.7 W/cm^2^	Wearable, spatiotemporally controllable, non-invasive	US-induced protein disaggregation & immune regulation	Alzheimer’s disease therapy
Tumor Diagnosis and Therapy	CWUS Patch (Zou et al., 2024) [16]	Fully integrated conformal wearable US patch system	2 mm thick	Tumor site, sonodynamic therapy	2.0 W/cm^2^ (intensity)	Good mechanical conformability, biocompatibility, portable, non-invasive	Focused US activation of sonosensitizer for ROS generation	Continuous SDT for deep-seated tumors
	wf-UMP (Xue et al., 2025) [47]	Lead-free US array + bioadhesive hydrogel + dissolvable microneedles	Microneedle height 600 μm, hydrogel thickness ~1 mm	Tumor, drug delivery & immunotherapy	10–120 Vpp driving voltage, 1.2 MHz center frequency	Flexible, wearable, bioadhesive, stable on dynamic tissue	US-enhanced drug delivery & immunomodulation	Cancer immunotherapy
	Tumor Monitoring & SDT (Siboro et al., 2024) [68]	Flexible TPU/HfO_2_ NPs sensor platform	Not specified	Tumor volume monitoring & sonodynamic therapy	1.0 W/cm^2^, 3 MHz	Flexible wearable, wireless data transmission	US volume monitoring + SDT (HfO_2_ as sonosensitizer)	Cancer theranostics & telemedicine
Other Applications	cUSB-Patch (Zhang et al., 2024) [74]	Sm/La-doped PMN-PT ceramic phased array	Overall thickness < 4.5 mm, single array 20.0 mm × 20.0 mm × 4.0 mm	Bladder volume monitoring	50 V, 3.5 MHz	Conformable, no manual operation/coupling gel needed (validated), wide field-of-view	Phased-array US volumetric imaging	Bladder volume monitoring (e.g., urinary retention)
	FES-Rehab System (Cao et al. 2025) [77]	Wearable musculoskeletal US + Functional Electrical Stimulation (FES)	Not specified	Muscle intent recognition, motor function assistance	US: 60 V driving voltage; FES: 10–21 mA, 30–40 Hz	Wearable integrated system, improved robustness & SNR	US-based intent recognition + synchronized FES	Post-stroke rehabilitation & motor function restoration
	Glucose Regulation (Yang et al., 2025) [80]	1–3 composite, Cu/PI electrodes, Ecoflex substrate	Not specified	Hepatic-pancreatic area, blood glucose regulation	986 kHz, 86.81 mW/cm^2^ (ISATA), 30% duty cycle, pulse repetition frequency of 2 kHz	Wearable patch + driver, biosafety validated in vivo	Low-intensity pulsed ultrasound therapy	Type 2 diabetes management

Note: BP, blood pressure; FPCB, flexible printed circuit board; ASIC, application-specific integrated circuit; HR, heart rate; VCSEL, vertical-cavity surface emitting laser; PLGA, poly (lactic-co-glycolic acid); SDT, sonodynamic therapy; PT, PbTiO_3_; SNR, signal-to-noise ratio; PI, polyimide.

## Data Availability

No new data were created or analyzed in this study. Data sharing is not applicable to this article.

## Abbreviations

The following abbreviations are used in this manuscript:

Aβ	β-Amyloid
AI	Artificial Intelligence
ASIC	Application-specific Integrated Circuit
BP	Blood Pressure
BTO	Barium Titanate
CHT	Chitosan
CMOS	Complementary Metal-oxide-semiconductor
CMUT	Capacitive Micromachined Ultrasonic Transducer
CNT	Carbon Nanotube
CWUS Patch	Conformal Wearable Ultrasound Patch
DS	Diclofenac Sodium
EPSC	Excitatory Postsynaptic Spontaneous Current
FAD	Familial Alzheimer’s Disease
FES	Functional Electrical Stimulation
FPCB	Flexible Printed Circuit Board
FUST	Flexible Ultrasound Transducer
GelMA	Gelatin Methacryloyl
HfO_2_	Hafnium Oxide
HR	Heart Rate
KA	Kainic Acid
KNN	Potassium Sodium Niobate
K2P channels	two-pore domain potassium channels
LIPUS	Low-intensity Pulsed Ultrasound
LTCC	L-type Calcium Channel
MEMS	Microelectromechanical Systems
MG-SOG	MscL-G22S-mediated Sonogenetics
MOF	Metal–organic Framework
MTX	Methotrexate
NP	Nanoparticle
PCL	polycaprolactone
PDMA	Piezoelectric-driven Microneedle Array
PDMS	polydimethylsiloxane
PEG	Polyethyleneglycol
PI	Polyimide
PLGA	Poly(lactic-co-glycolic acid)
PMN	Pb(Mg_1/3_Nb_2/3_)O_3_
PMUT	Piezoelectric Micromachined Ultrasonic Transducer
PT	PbTiO_3_
PV	Parvalbumin
PVDF	Polyvinylidene Fluoride
PWI	Pulse Wave Imaging
PZT	Lead Zirconate Titanate
RA	Rheumatoid Arthritis
RCT	Randomized Controlled Trial
REP	Repaglinide
rGO	Reduced Graphene Oxide
ROS	Reactive Oxygen Species
SDT	Sonodynamic Therapy
SE	Status Epilepticus
SLN	Solid Lipid Nanoparticle
sn-CMUT	Silicon Nanopillar Capacitive Micromachined Ultrasonic Transducer
SST	Somatostatin
TDDS	Transdermal Delivery System
TPU	Thermoplastic Polyurethane
TUT	Transparent Ultrasonic Transducer
UsoP	Ultrasound Patch
VCSEL	Vertical-cavity Surface Emitting Laser
wf-UMP	Wearable Flexible Ultrasound Microneedle Patch
WUS	Wearable Ultrasound Sensor
